# Herbal and Natural Treatments for the Management of the Glaucoma: An Update

**DOI:** 10.1155/2023/3105251

**Published:** 2023-11-17

**Authors:** Livio Vitiello, Luigi Capasso, Giovanni Cembalo, Ilaria De Pascale, Roberto Imparato, Maddalena De Bernardo

**Affiliations:** ^1^Eye Unit, “Luigi Curto” Hospital, Azienda Sanitaria Locale Salerno, Polla, 84035 Salerno, Italy; ^2^Eye Unit, “Ospedale del Mare” Hospital, Azienda Sanitaria Locale Napoli 1 Centro, Naples 80147, Italy; ^3^Eye Unit, Department of Medicine, Surgery and Dentistry “Scuola Medica Salernitana”, University of Salerno, Baronissi, 84081 Salerno, Italy; ^4^Eye Unit, “Ramazzini” Hospital, Azienda Unità Sanitaria Locale Modena, Carpi 41012, Italy; ^5^Eye Unit, Azienda Unità Sanitaria Locale Valle d'Aosta, Aosta 11100, Italy

## Abstract

Glaucoma causes the degeneration of the retinal ganglion cells (RGCs) and their axons, inducing a tissue reshaping that affects both the retina and the optic nerve head. Glaucoma care especially focuses on reducing intraocular pressure, a significant risk factor for progressive damage to the optic nerve. The use of natural treatments, such as herbs, vitamins, and minerals, is becoming increasingly popular today. While plants are a rich source of novel biologically active compounds, only a small percentage of them have been phytochemically examined and evaluated for their medicinal potential. It is necessary for eye care professionals to inform their glaucoma patients about the therapy, protection, and efficacy of commonly used herbal medicines, considering the widespread use of herbal medicines. The purpose of this review is to examine evidence related to the most widely used herbal medicines for the management and treatment of glaucoma, to better understand the potential benefits of these natural compounds as supplementary therapy.

## 1. Introduction

Glaucoma is the leading cause of irreversible blindness worldwide [1]. The disease has a complex etiology and a wide range of risk factors; all these factors can be associated with death of retinal ganglion cells (RGCs) and to tissue remodelling affecting both the retina and the optic nerve head. Furthermore, the optic nerve becomes thinner, and the lateral geniculate ganglion cells partly vanish over time. Moreover, the blood flow tends to be reduced in different ocular tissues in glaucoma patients, thus contributing to neuronal damage [2]. Reducing intraocular pressure (IOP), a substantial risk factor for progressive optic nerve injury, is a major focus of glaucoma treatment. Nevertheless, it is obvious that IOP is only one of several variables that lead to glaucoma pathophysiology [3].

Neuroprotection is a fast-growing area of investigation. This topic is really challenging because it is a new treatment option for frustrating disorders that may worsen despite optimal therapy, as in the case of glaucoma [1]. One of the first medicinal plants used for glaucoma was pilocarpine, a parasympathomimetic alkaloid extracted from a South American shrub, Pilocarpus microphyllus [4].

Considering the extensive usage of herbal medications, eye care professionals should educate their glaucoma patients about the treatment, safety, and efficacy of these drugs. In fact, nowadays, using natural remedies like herbs, vitamins, and minerals to treat ocular disorders is becoming more and more popular [5–10]. Alternative medications have a $109 billion global market and are commonly used by up to 52 percent of the general population [11, 12]. Despite being a plentiful source of new biologically active compounds, only very few plants have been phytochemically analyzed and evaluated for their potential as medicines [13, 14].

Today, while active compounds continue to be extracted from medicinal plants by the pharmaceutical industry, standardized techniques are used to achieve this aim. In fact, in the United States, the Food and Drug Administration (FDA) enforces these uniform procedures and controls prescription medications, but not complementary medicines, such as herbal medicines and dietary supplements [10]. Considering that glaucoma induces a RGC degeneration, the neuroprotection has gained progressively more interest as a new strategy to prevent or delay the progression of the morphological and functional glaucomatous damages. Since the use of natural treatments to treat several pathologies is becoming more popular, claiming for efficacy and reduced adverse events, it could be interesting to deeply examine the possible use of herbal medicines applied to the glaucoma management.

For this reason, the purpose of this review is to assess evidence related to the most widely used herbal medicines in the management and treatment of glaucoma through an overview of the scientifically published literature and to better understand the potential benefits of these natural compounds as supplementary therapy for glaucoma.

## 2. Materials and Methods

We carried out a search on the PubMed and Scopus medical databases. To gain a broader perspective and comprehension of the problem, a preliminary generic Google search was also performed. The database search strategy was formulated around the term “glaucoma” and several other terms regarding medicinal herbs, natural compounds, and phytotherapy (“Baicalein”, “Baicalin”, “Wogonin”, “Ashawagandha”, “Withania”, “phytotherapy” “Saffron”, “Crocus sativus”, “Bilberry”, “Anthocyanin”, “Erigeron”, “Curcuma”, “Curcumin”, “Ginkgo Biloba”, “Salvia miltiorrhiza”). The search terms were selected after considering the available literature and/or gathered from linked bibliographies. Only full articles and case reports were reviewed. Duplicate and unrelated papers were excluded. Bibliographies from the initial searches were also manually searched for additional inclusions.

## 3. Results

### 3.1. Ginkgo biloba

The history of Ginkgo biloba began around 250 million years ago, and it is a member of the earliest order of true trees. These trees are commonly planted in Asian countries, especially in China and Japan, and the seeds are used to treat a wide range of systemic disorders [15, 16]. Among its properties, vasoactive effects [17, 18], hemorheological regulation [19, 20], anti-inflammatory [21–23], and antioxidative capacities [24] have been demonstrated. The extract from Ginkgo biloba leaves, known as Ginkgo biloba extract 761 (EGb761), has been demonstrated to be helpful for dementia and cognitive impairment in modern medicine [25]. Researchers have examined Ginkgo biloba for glaucoma due to the molecular and mechanical parallels between Alzheimer's disease and glaucoma [26]. The significance of mitochondrial dysfunction in the glaucoma etiology has been shown in a number of investigations [27]. Only antioxidants with mitochondrial penetration potential can serve as effective neuroprotective agents, and some compounds in Ginkgo, such as polyphenolic flavonoids, may theoretically reduce oxidative stress in the mitochondria and shield RGCs [16, 28, 29].

Recently, one in vitro study revealed a significative reduction of damages induced by hydrogen peroxide on RGCs as well as in an animal model [30]. In another in vivo study, the effects of Ginkgo biloba extract (GBE) on elevated IOP and RGC density were evaluated, revealing that both pretreatment and early posttreatment with EGb761 have a successful neuroprotective effect in a rat model of chronic glaucoma [31].

The therapy with GBE in a group of patients with normal tension glaucoma (NTG) seems not to have any impact on the mean defect or contrast sensitivity on the 24-2 SITA standard visual field [26]. In this study, patients who had just received an NTG diagnosis had two 4-week treatment phases, each followed by an 8-week washout period, showing no significant improvement in the management of the disease [32].

Conversely, in another retrospective study, forty-two eyes of 42 patients with treated NTG were assessed. They received 80 mg GBE twice daily, and they had at least 5 visual field tests using the Humphrey Visual Field Analyzer for more than a 4-year period before and after GBE treatment. Indexes such as mean deviation, pattern standard deviation, and visual field index were measured, showing that GBE administration slowed the progression of visual field damage in NTG patients [33].

For this reason, considering the controversial results emerging from studies in the literature, more studies are needed to establish the actual usefulness of GBE in glaucoma therapy and management. Furthermore, it is crucial to consider that none of the studies currently published in the literature on Ginkgo biloba deals with the treatment and management of primary open-angle glaucoma (POAG), which accounts for nearly 90% of glaucoma cases.

### 3.2. Scutellaria baicalensis Georgi Derivates

Scutellaria baicalensis Georgi is a medicinal herb widely available in several East Asian countries and in China. Since ancient times, its dried root, known as Scutellariae radix, has been used in the therapeutic treatment of several illnesses, including hepatitis, hepatoma, leukemia, and hyperlipidemia. Additionally, this medicinal plant can reduce blood pressure, capillary permeability, and serum cholesterol levels [34–36].

Baicalin (5,6-dihydroxy-7-O-glucuronide), wogonin (5,7-dihydroxy-8-methoxyflavone), and baicalein (5,6,7-trihydroxyflavone) are the principal bioflavones extracted from this radix. They have a broad spectrum of bioactivity, including antioxidant, anticancer, anti-inflammation effects, and neuroprotection [37–42]. These bioflavones have been demonstrated to exert their effects through different pathways, such as inhibiting nitric oxide production in macrophages and the gene expression of lipopolysaccharide inducible nitric oxide synthase, suppressing cyclooxygenases-2 gene expression and prostaglandin E2 production, and downregulating different proinflammatory mediators and cytokines [43–47]. Particularly, they are capable of reducing interleukin-8 (IL-8), which is generally increased in POAG patients [48].

Concerning their antioxidant activities, it has been proved that these compounds are able to scavenge reactive oxygen species and to prevent their production by inhibiting xanthine oxidase [49, 50]. Moreover, they can act as iron chelators and inhibitors of the Fenton reaction [51].

The neuroprotective effects against oxidative stress of both baicalin and baicalein have been proved in RGCs. An in vitro study carried out in an immortalized RGC line (RGC-5) demonstrated that baicalein effectively rescued RGCs in three different oxidative stress models [52]. Equally, in the RGC-5, baicalin showed effective, dose-dependent protective effects against several different oxidative insults, such as serum deprivation, hydrogen peroxide treatment, and light exposure [53]. Therefore, these results suggest that baicalin and baicalein have a strong capacity to protect RGCs from oxidative stress, assuming their possible function in reducing RGC loss in glaucoma.

In addition, an in vitro study by Gong and Zhu on human trabecular meshwork cells [54] showed how baicalin was able to reduce intracellular reactive oxygen species and increase cell survival, inhibiting the proinflammatory factor interleukin-1*α* (IL-1*α*) and endothelial leucocyte adhesion molecule-1 (ELAM-1) production, decreasing activities of senescence-associated beta-galactosidase (SA-*β*-gal), and lowering levels of carbonylated protein.

For flavonoid-induced protection against oxidative stress in brain cells, three separate pathways have been identified: raising glutathione levels, removing reactive oxygen species, and blocking Ca2+ influx [55].

Regarding the role of wogonin in preventing RGC loss in glaucoma, a study by Xu et al. [56] on the optic nerve crush model proved its ability to suppress inflammatory responses and rescue RGC death. In fact, wogonin can reduce RGC loss and inhibit RGC apoptosis, decreasing the level of toll-like receptor 4 (TLR4 expression), nuclear factor kappa-light-chain-enhancer of activated B cells-P65 (NF-*κ*B-P65), and NF-*κ*B-P65 phosphorylation. For this reason, all these bioflavones could be considered possible therapeutic agents for the glaucoma treatment and management.

Finally, although the bioflavones extracted from the Scutellaria baicalensis Georgi have proven their ability in preventing RGC loss in glaucomatous animal models in vitro, they have not been used yet in vivo glaucoma patients, and, consequently, their efficacy in the glaucoma management in human subjects needs to be further demonstrated.

### 3.3. Crocus sativus L. (Saffron)

The dried stigmas of the stemless plant Crocus sativus L. flower compose the saffron, which is mostly used in cooking as a flavouring and colouring spice. Picrocrocin, safranal, crocetin, and crocin are the pharmacologically active components of saffron [57, 58].

Saffron or its active ingredients may possibly have neuroprotective, antioxidant, anti-inflammatory, antidepressive, anxiolytic, anticonvulsant, antiatherogenic, hypolipidemic, antihypertensive, and even antitumor actions, according to growing data from pharmacological research [59–61].

Crocin and crocetin were found to be able to inhibit RGC death in mouse and rat models of ischemia/reperfusion [62–64].

The decrease in the number of positive terminal deoxynucleotidyl transferase dUTP nick end labelling (TUNEL) cells and 8-hydroxy-2-deoxyguanosine positive cells, as well as the elevated phosphorylation levels of c-Jun, c-Jun N-terminal kinases (JNK), p38, and NF-B following retinal damage, are all examples of mechanisms that may be responsible for the potential neuroprotective effects against ischemic damage [62].

Recently, in a mouse model of glaucoma, the neuroprotective and anti-inflammatory effects of the saffron extract have been confirmed [65]. In fact, in both the ocular hypertension (OHT) eye and the normotensive fellow eye, saffron extract therapy decreased the number of microglial cells and the symptoms of microglial activation. Additionally, supplementing with saffron extract partially corrected the downregulation of Purinergic Receptor P2RY12 brought on by the IOP increase [65]. Immediately after the injury, P2RY12 expression is upregulated. On the other hand, a few hours later, P2RY12 expression is downregulated and disappears in highly active microglial cells [66]. As a result, this finding suggests that saffron extract may reduce inflammation by controlling P2RY12 expression [65]. Finally, in this model, it has also been shown that saffron may stop OHT eyes from losing considerable amounts of transcription factor Brn3a+ RGCs [65]. The antioxidant and anti-inflammatory properties of the saffron extract could be responsible for this substance's neuroprotective benefits [65].

The few clinical investigations that have been conducted on individuals with POAG have examined any potential ocular hypotensive effects, and their findings have been conflicting. Saffron supplementation (1 g twice a week) in a randomized controlled pilot study did not seem to have an impact on IOP in the near term (1 month) [67]. However, according to a different pilot study carried out on 34 clinically stable POAG patients, oral saffron supplementation (30 mg daily for one month) can considerably lower IOP after three weeks. Though, this effect is reversed after a four-week washout period [68].

### 3.4. Coleus forskohlii

The plant Coleus forskohlii has been used in Ayurvedic and Hindu traditional medicine for a very long time. Its root, which has historically been used in medicine, includes forskolin, the active constituent.

Through the activation of the enzyme adenylate cyclase, forskolin's main mechanism of action is to enhance cyclic adenosine monophosphate (cAMP) and cAMP-mediated activities [69].

Forskolin is able to inhibit basophil and mast cell degranulation and histamine release [70], lower blood pressure [71] and IOP [72], inhibit platelet aggregation [73], and promote vasodilation [74], bronchodilation [75], and thyroid hormone secretion [76], as well as stimulate lipolysis in fat cells [77].

Forskolin also has the ability to block the binding of platelet-activating factor (PAF), irrespective of its ability to stimulate cAMP production. Additionally, forskolin can reduce glucose transfer in erythrocytes, adipocytes, platelets, and other cells [78]. It also appears to have an impact on different membrane transport proteins [78].

Several studies using topical forskolin applications to decrease IOP have been carried out, with contrasting results.

IOP was significantly reduced in a dose-dependent manner in the eyes of normal rabbits using solutions of 2%, 1%, and 0.5% forskolin. These effects peaked in 2-3 hours and lasted up to 10 hours [79]. On the other hand, glaucomatous monkeys treated for two days with a 1% forskolin solution failed to appreciably lower IOP [80]. However, Caprioli and Sears found that giving rabbits, monkeys, and people a topical 1% forskolin solution significantly reduced their IOP. This effect started at one hour after application and persisted for at least five hours [81]. Forskolin has also been demonstrated to be beneficial at lowering IOP and reducing aqueous outflow in human trials with only healthy volunteers.

In a randomized, crossover experiment, Meyer et al. evaluated the effects of 1% forskolin against placebo on 10 healthy volunteers. IOP decreased in the first trial in both the forskolin and placebo groups, which was attributable to the local anesthetic oxybuprocaine. In the second experiment, forskolin substantially reduced IOP compared to placebo when proxymetacaine was employed as the topical anesthetic [82]. Conversely, one dosage of a 1% forskolin solution had no impact in a different trial on 20 healthy participants, but two instillations spaced out by five minutes significantly reduced IOP and aqueous flow rate [83].

However, a research by Brubaker et al. found no evidence that forskolin could significantly reduce flow rate in a group of 15 healthy volunteers who received one dosage of 1% forskolin in each of three circumstances: during the day, while sleeping at night, and after receiving timolol pretreatment [84].

Clinical research on the use of forskolin in glaucoma patients is scarce, despite its topical use in healthy humans and animals that seems promising. Mutolo et al. studied twenty-two POAG patients already under treatment with IOP-lowering medications to determine the effects of a food supplement containing forskolin; homotaurine; carnosine; folic acid; vitamins B1, B2, and B6; and magnesium over the course of a 12-month period. They found a further IOP decrease and a foveal sensitivity and pattern electroretinogram amplitude improvement in treated patients with this food supplement, also showing a short-term neuroactive effect [85].

Due to a lack of information on the potential effects of forskolin on glaucomatous patients, especially if orally administered, further studies on this important topic are needed.

### 3.5. Vaccinium myrtillus (Bilberry)

Anthocyanins derived from Vaccinium myrtillus or bilberry are increasingly being used in ophthalmology. In fact, vascular tissues and eyes are particularly responsive to flavonoid anthocyanosides [86]. Strong antioxidant properties [87]; decreased capillary permeability and fragility [88]; collagen fiber stabilization and collagen biosynthesis promotion [89]; prevention of the release and synthesis of proinflammatory compounds such as histamine, prostaglandins, and leukotrienes [90]; inhibition of platelet aggregation [91]; and blood glucose level decrease [92] are just a few of the anthocyanins' mechanisms of action. Although there have not been many studies on this challenging topic, anthocyanins have shown promising actions in the glaucoma treatment and management.

In a recent study on mice [93], anthocyanins in bilberry extract (100 mg/kg/day or 500 mg/kg/day) were administered orally, and the expression levels of various molecular chaperones and RGC survival were appraised. This study showed that oral bilberry extract administration could suppress RGC death and increase glucose-regulated protein 78 (Grp78) and Grp94 protein levels, an effect which may underlie the neuroprotective effect of bilberry extract after optic nerve damage.

Caselli [94] evaluated eight patients with glaucoma that received a single dose of 200 mg anthocyanosides from bilberry, with significant improvements at the electroretinography.

In another study [95], a retrospective analysis was carried out by a chart review of 332 NTG subjects (209 men and 123 women), who were treated with anthocyanins (*n* = 132), GBE (*n* = 103), or no medication (controls, *n* = 97). Before and after therapy, the Humphrey Visual Field (HVF) test, best-corrected visual acuity, IOP, blood pressure, and fasting blood glucose levels were measured. HVF mean deviation is improved following anthocyanin and GBE therapy, indicating that anthocyanins and GBE may be useful in enhancing visual function in some NTG subjects.

Finally, Gizzi et al. [96] assessed 88 patients affected by ocular hypertension who were monitored for 12 weeks in a supplement registry. The patients were divided into three study groups by the authors: the first group received latanoprost plus Mirtogenol® (80 mg of bilberry extract, Mirtoselect®, plus 40 mg of Pycnogenol®), the second group received latanoprost alone, and the third group received dorzolamide-timolol plus Mirtogenol®. IOP, retinal blood flow, Zinn-Haller circle perfusion, and oxidative stress were evaluated. According to the study, all treatment groups showed statistically significant improvements in retinal microcirculation and IOP throughout the course of the trial, with a little more pronounced effect in the first group. In addition, Mirtogenol® users exhibited better perfusional patterns than patients who just used latanoprost. Additionally, supplemented participants showed a decrease in oxidative stress. In order to achieve normal IOP and ophthalmic microcirculatory parameters, supplement with Mirtogenol® in addition to local ocular therapies might be considered both safe and helpful.

To further understand the possible significance of anthocyanins in the management and therapy of glaucoma, more studies should be carried out in the future.

### 3.6. Ribes nigrum L. (Black Currant Fruits)

As bilberry, black currant fruit is rich in anthocyanins and is commonly consumed worldwide too. Black currants contain only four different anthocyanins, a simpler composition than bilberries. We found some published papers about black currant anthocyanin intake related to glaucoma progression.

Ohguro et al. [97] performed a randomized, placebo-controlled, double-masked trial concerning oral black currant anthocyanins (BCACs) and POAG progression in 38 patients in treatment with antiglaucomatous drops. BCACs (50 mg/day, *n* = 19) or their placebos (*n* = 19) were orally administered once daily for a 24-month period. The authors found a statistically significant improvement in ocular blood flow and in the visual field in the BCAC-treated group (oral administration of 50 mg/day). Nevertheless, they did not find significant changes in other ocular parameters, as in IOP values, during the study period.

In a randomized, placebo-controlled, double-masked 24-month trial on POAG patients (*n* = 19), Yoshida et al. [98] found that, after BCAC intake (2 capsules, 50 mg/day), the serum endothelin-1 (ET-1) concentration in POAG patients was significantly increased to levels similar to those of healthy subjects (*n* = 20). On the other hand, ET-1 values for placebo-treated patients remained lower, similar to the baseline.

Once more, Ohguro et al. [99] carried out a placebo-controlled, double-masked, crossover study in 21 POAG patients (BCACs, *n* = 12; placebo, *n* = 9) treated with a single antiglaucomatous drug and 12 healthy subjects treated once daily with oral BCACs (50 mg). They found a statistically significant decline in mean IOP values (at 4 weeks, *p* = 0.039) not only in healthy participants but also in POAG patients using BCACs (*p* = 0.027) after a 24-month period.

All these results suggest that oral BCAC intake may induce an improvement in ocular blood flow and a decrease in IOP levels both in healthy and POAG patients already treated with antiglaucomatous drugs.

### 3.7. Erigeron breviscapus

Erigeron breviscapus is an herbal medicine spread in Yunnan Province, well known as a Chinese medicinal plant for heart disease. Several studies have shown that it can improve blood circulation and reduce thrombotic events [100, 101].

We found a published article which correlates this plant intake to improvement in IOP values. In fact, Zhong et al. [102] performed a randomized, double-blind, clinical trial on POAG patients, with visual field defects and with a postsurgical IOP of less than 18 mmHg (*n* = 40 eyes), administering orally Erigeron tablets and placebo tablets, 2 tabs three times in a day for 6 months. Erigeron-treated group showed a significant decrease in mean defect and a significant increase in mean sensitivity (*p* < 0.05) after 6 months, while the placebo group did not show any significant changes. These results could suggest that Erigeron breviscapus intake may have a stimulating effect in glaucoma patients with a controlled range IOP.

### 3.8. Salvia miltiorrhiza

Salvia miltiorrhiza is a traditional botanical Chinese medicine that, when administered intravenously, seems to improve microcirculation [103]. For this reason, this extract may have neuroprotective effects on the retina in progressive glaucoma.

In a study carried out by Zhu and Cai on 36 pigmented rabbits, this botanical compound was found to protect the optic nerve from the damage related to increased IOP [104].

In a different study using a rat glaucoma model, Zhu et al. [105] reported the outcomes from 20 male Sprague Dawley rats that had their aqueous outflow blocked by laser radiation, compared to 10 control rats. In the laser-induced glaucoma model, they discovered that Salvia miltiorrhiza extract was unable to stop the IOP rise, but the therapy reduced cell death as the glaucoma advanced, suggesting its neuroprotective properties against the disease [105].

In a human study [106], a 2 g/mL solution of Salvia miltiorrhiza alone or in combination with other Chinese herbs was administered daily through intramuscular injection to 121 individuals (153 eyes) affected by moderate or late-stage POAG and under antiglaucomatous therapy. After 30 days, 49.7% of the eyes' visual field and 43.8% of the eyes' visual acuity had both improved. The four herbal remedies did not differ significantly from one another, but there was a statistically significant improvement compared to the untreated eyes. Following the monthly treatment, 19 eyes were reassessed 7–30 months later, and the results showed that 73.7% of the eyes had maintained or improved their visual fields, assuming a potential long-term effect of this herbal remedy.

## 4. Discussion

Glaucoma is a particularly complex disease, for both diagnosis and management [107–114].

Neuroprotective therapy is another innovative method for managing glaucoma, and its goal is to stop or delay RGC degeneration and death [8].

Traditional Chinese medicine, already for about 50 years, has always focused on the beneficial and healing effects of natural compounds for the treatment of various diseases [115–118].

For this reason, in recent years, several natural compounds have been analyzed to better understand their potential beneficial effects in the treatment of ocular neurodegenerative diseases, such as glaucoma and age-related macular degeneration [5–10].

Possible neuroprotective substances found in nature, such the bilberry and GBE, have received growing attention for this therapeutic approach [8]. Many of these compounds have been shown to increase choroidal and retinal circulation and facilitate retinal recovery after ischemic insult in animals [10]. However, to demonstrate the usefulness of neuroprotective medicines against glaucoma, including medicinal herbs, high-quality data is currently missing.

Concerning GBE, it has been shown that it has neuroprotective properties that guard against RGC degradation in glaucoma. Several mechanisms have been clarified using modern scientific tools, but many more properties still require additional research [9].

Regarding saffron, its chemical analysis has revealed that it contains more than 150 volatile and aroma-producing chemicals, as well as a number of nonvolatile biologically active ingredients, such as crocetin and zeaxanthin carotenoids, and other alpha- and beta-carotenes [6]. Crocetin, a carotenoid dicarboxylic acid, and crocin appear to be the most powerful antioxidant components of saffron. In fact, the crocin's antiapoptotic activity and improved oxygen diffusivity in mammalian tissues are already known [6]. According to research by Kanakis et al. [119], saffron metabolites directly attach to DNA and cause it to partially conform to beta-DNA, shielding the cell from damage.

Furthermore, it has been demonstrated that saffron has anti-inflammatory properties, such as the ability to inhibit tissue necrosis factor. For this reason, the unusual properties of saffron components may employ several distinct mechanisms of action, ranging from direct regulation of gene expression to antioxidant activity that could be crucial for neuroprotection [6].

This review showed that, in addition to GBE and saffron, many other natural compounds may be useful in preserving RGCs and reducing IOP, thus demonstrating a promising and helpful role in the glaucoma therapy and management. In fact, all analyzed herbal medicines have been proven to be effective or have shown encouraging effects in neuroprotection reducing RGC death, except for Coleus forskohlii.

On the other hand, only Vaccinium myrtillus and Ribes nigrum showed positive and promising effects on decreasing IOP, while all other natural compounds showed either no effects or conflicting results in the various studies, in particular Crocus sativus and Coleus forskohlii (Tables 1 and 2).

From our analysis, according to the previously published literature [5–10], it seems that the main beneficial effects of these natural compounds can be attributed to their antioxidant and anti-inflammatory properties, capable of exerting a neuroprotective effect, especially on RGCs.

The present review has several limitations, including the too small number of papers embedded for the predetermined analysis, especially considering the clinical studies performed on human models and glaucomatous patients. In fact, several studies were performed only on healthy volunteers, while no study included both POAG and NTG patients to compare the therapeutic effects of these herbal treatments.

In addition, all included papers have a small sample size, which makes the obtained data less meaningful.

## 5. Conclusions

In conclusion, the natural compounds analyzed in this review appear to show promising effects, especially on neuroprotection, while they seem to be less effective on IOP decrease. However, further studies are needed to better understand how these herbal compounds can possibly be incorporated into glaucoma therapeutic protocols.

## Figures and Tables

**Table 1:** Main-demonstrated biological properties of the discussed herbal compounds.

Herbal compounds	Biological properties
*Ginkgo biloba*	Vasoactive effects Hemorheological regulation Anti-inflammatory effects Antioxidative capacities Neuroprotection

*Scutellaria baicalensis Georgi*	Blood pressure, capillary permeability, and serum cholesterol level reduction Antioxidative effects Antitumor capacities Anti-inflammatory effects Neuroprotection

*Crocus sativus L.*	Antioxidative effects Anti-inflammatory effects Antidepressive, anxiolytic, and anticonvulsant capacities Antiatherogenic, hypolipidemic, and antihypertensive effects Antitumor actions Neuroprotection

*Coleus forskohlii*	Basophil and mast cell degranulation inhibition Histamine release inhibition Blood pressure and intraocular pressure reduction Platelet aggregation inhibition Vasodilation, bronchodilation, and thyroid hormone secretion promotion lipolysis in fat cell stimulation

*Vaccinium myrtillus*	Strong antioxidant properties Capillary permeability and fragility reduction Collagen fiber stabilization and collagen biosynthesis promotion Anti-inflammatory effects Platelet aggregation inhibition Blood glucose level reduction

*Ribes nigrum L.*	Antioxidant properties Capillary permeability and fragility reduction Collagen fiber stabilization and collagen biosynthesis promotion Anti-inflammatory effects Platelet aggregation inhibition Blood glucose level reduction

*Erigeron breviscapus*	Blood circulation improvement Antithrombotic effects

*Salvia miltiorrhiza*	Vascular microcirculation improvement

**Table 2:** Summary of the main effects of the discussed herbal compounds on retinal ganglion cells and intraocular pressure.

Herbal compounds	In vitro or animal studies	Human studies	Effects on RGCs	Effects on IOP
*Ginkgo biloba*	2	2	Possible reduction of the damage induced by oxidant agents both in animal models and in NTG patients [30–33] No data on POAG patients	Not demonstrated

*Scutellaria baicalensis Georgi*	4	—	Strong capacity to protect RGCs from oxidative stress in animal and in vitro models [52–54, 56] No data on human models	Not demonstrated

*Crocus sativus L.*	1	2	Neuroprotective effects derived from antioxidant and anti-inflammatory effects both in animal models and in POAG patients [65] No data on NTG patients	Conflicting results from human studies on POAG patients [67, 68] No data on NTG patients

*Coleus forskohlii*	3	4	Not demonstrated	Conflicting results from studies on animal models, healthy people, and POAG patients [79–85] No data on NTG patients

*Vaccinium myrtillus*	1	3	Neuroprotective effects through retinal microcirculation improvement both in animal and human models [93]	Slight IOP reduction if administered orally in patients with ocular hypertension [94–96] No data on POAG and NTG patients

*Ribes nigrum L.*	—	3	Neuroprotective effects through ocular blood flow improvement in POAG patients [97, 98] No data on NTG patients	IOP decrease both in healthy people and POAG patients [99] No data on POAG patients

*Erigeron breviscapus*	—	1	Stimulating effects in POAG patients with IOP in a controlled range [102] No data on NTG patients	Not demonstrated

*Salvia miltiorrhiza*	1	1	Neuroprotective effects on animal model and POAG patients [104, 105] No data on NTG patients	Not demonstrated
